# Elevated Neutrophil to Lymphocyte Ratio Associated With Increased Risk of Recurrent Vascular Events in Older Minor Stroke or TIA Patients

**DOI:** 10.3389/fnagi.2021.646961

**Published:** 2021-04-20

**Authors:** Ka Lung Chan, Xueyan Feng, Bonaventure Ip, Shangmeng Huang, Sze Ho Ma, Florence S. Y. Fan, Hing Lung Ip, Li’an Huang, Vincent C. T. Mok, Yannie O. Y. Soo, Thomas W. Leung, Xinyi Leng

**Affiliations:** ^1^Department of Medicine and Therapeutics, The Chinese University of Hong Kong, Hong Kong, China; ^2^Department of Neurology, The First Affiliated Hospital, Jinan University, Guangzhou, China; ^3^Department of Neurology, Beijing Tiantan Hospital, Capital Medical University, Beijing, China; ^4^Department of Geriatrics, The First Affiliated Hospital of Xiamen University, Xiamen, China

**Keywords:** minor stroke, transient ischemic attack, neutrophil to lymphocyte ratio, inflammation, prognosis

## Abstract

**Background:**

The risk of recurrent stroke following a minor stroke or transient ischemic attack (TIA) is high, when inflammation might play an important role. We aimed to evaluate the value of neutrophil to lymphocyte ratio (NLR) in predicting composite cardiovascular events in patients with minor stroke and TIA.

**Methods:**

Consecutive patients with acute minor stroke or TIA admitted within 24 h of symptoms onset during a 5-year period in a prospective stroke registry were analyzed. We calculated the NLR dividing absolute neutrophil count by absolute lymphocyte count tested within 24 h of admission. NLR ≥4th quartile was defined as high NLR. A composite outcome was defined as stroke, acute coronary syndrome or vascular death within 1 year. We investigated associations between NLR and the composite outcome in univariate and multivariate analyses, among all patients and in those aged over 60 years (i.e., older patients).

**Results:**

Overall, 841 patients (median age 68 years; 60.4% males) were recruited. No significant independent association was found between NLR and the composite outcome in multivariate analysis in the overall cohort. Among the 612 older patients (median age 73 years; 59.2% males), the median NLR was 2.76 (interquartile range 1.96−4.00) and 148 (24.2%) patients had high NLR. The composite outcome occurred in 77 (12.6%) older patients, who were more likely to have a high NLR (39.0% versus 22.1%; *p* = 0.001) than those without a composite outcome. In multivariate logistic regression, high NLR (adjusted odds ratio 2.00; 95% confidence interval 1.07−3.75; *p* = 0.031) was independently associated with the composite outcome in older patients.

**Conclusion:**

In older (aged ≥60 years) patients with acute minor stroke or TIA, a higher NLR, a marker of systemic inflammation that can be easily obtained in routine blood tests, is an independent predictor of subsequent cardiovascular events.

## Introduction

Transient ischemic attack (TIA) and minor ischemic stroke account for up to 65% of all acute ischemic cerebrovascular events (von Weitzel-Mudersbach et al., 2013). Although they are mostly non-disabling, subsequent recurrent strokes may be disabling. The risk of recurrent stroke following a TIA or minor stroke is high. For instance, in the Clopidogrel in High-Risk Patients with Acute Non-disabling Cerebrovascular Events (CHANCE) trial, 8.2% of minor stroke or high-risk TIA (ABCD2 score of 4 or higher) patients suffered another stroke within 90 days, when receiving early initiated aspirin and clopidogrel treatment for 21 days and clopidogrel for day 22−90 (Wang et al., 2013). The more recently published multicenter, worldwide TIAregistry.org project revealed annual risks of stroke and all cardiovascular events of 5.1 and 6.2%, respectively, among 4,789 TIA or minor stroke patients, despite faster acute management and more efficient secondary prevention by dedicated TIA services in the participating centers in recent years (Amarenco et al., 2016). There have been a few well-established risk factors for recurrent stroke in such patients, such as aging, history of hypertension and poorly controlled blood pressure, diabetes, prior TIA, as well as the clinical features of the symptoms and some imaging markers, which has been incorporated in risk stratification scores, such as the ABCD2 (age, blood pressure, clinical features, duration of symptoms, and history of diabetes), ABCD3 (ABCD2 elements plus dual TIAs), and ABCD3-I (ABCD3 elements plus acute diffusion-weighted imaging hyperintensity and ≥50% ipsilesional carotid artery stenosis) scores (Merwick et al., 2010).

In addition to these factors, inflammation might play an important role in the relapse following an ischemic stroke or TIA. Necrotic cells, cell death debris, and increased reactive oxygen species resulted from brain ischemia could activate robust inflammation, including attracting infiltrating leukocytes from circulating blood into the brain tissue (Jin et al., 2010). Various inflammatory markers have been associated with the risk of future vascular events after ischemic stroke (Castillo et al., 2009; Whiteley et al., 2011). The neutrophil to lymphocyte ratio (NLR), calculated as the ratio of absolute neutrophil count and absolute lymphocyte count, has been suggested as an inflammation marker with prognostic value in patients with acute ischemic stroke or acute coronary syndrome (ACS) (Tamhane et al., 2008; Gul et al., 2014). Recent studies indicated that among acute ischemic stroke patients, NLR was independently associated with recurrent stroke, unfavorable functional outcome, stroke-associated pneumonia and hemorrhagic transformation following thrombolysis or endovascular therapy (Guo et al., 2016; Xue et al., 2017; Nam et al., 2018; Pikija et al., 2018). Also, early NLR was associated with unfavorable functional outcome within 90 days in patients with acute minor stroke or TIA (Luo et al., 2020). However, data are limited for the prognostic value of NLR in predicting long-term risks of vascular events in patients with minor stroke or TIA. In the present study, we therefore aimed to investigate the association between NLR and a composite outcome of stroke, ACS and vascular death within 1 year after a minor stroke or TIA.

## Materials and Methods

### Study Design and Subjects

We analyzed data collected prospectively for the stroke registry at Prince of Wales Hospital, Hong Kong SAR, which included all stroke and TIA patients in the catchment area presented at the hospital. All patients in the registry were regularly followed up at a neurology outpatient clinic, when recurrent cerebral ischemic events and other events were recorded. Consecutive, adult patients with acute minor ischemic stroke or TIA admitted within 24 h of symptoms onset, from January 1, 2011 to December 31, 2015, were recruited to the current study. Minor ischemic stroke was defined by a National Institute of Health Stroke Scale (NIHSS) of ≤3 upon admission. TIA was defined as a transient episode of neurological dysfunction caused by focal brain ischemia, without acute infarction in computed tomography (CT) or diffusion-weighted magnetic resonance imaging (DWI). Patients with any of the following conditions were excluded: (1) there was no blood cell counts data; (2) a history of cancer; (3) severe hepatic or renal diseases; (4) autoimmune diseases or use of steroids or immunosuppressants; (5) any infection within 2 weeks before the index stroke/TIA onset or during hospitalization (as noted in the clinical records, or taking antibiotics); (6) hematologic disorders; and (7) major trauma or severe bleeding. The study was approved by the Joint Chinese University of Hong Kong–New Territories East Cluster Clinical Research Ethics Committee, and consent was waived with no additional contact with the patients for the research purpose.

We collected patients’ baseline data including demographics (age, sex), history of common vascular risk factors [hypertension, diabetes, dyslipidemia, atrial fibrillation (AF), and smoking status], history of ischemic heart disease, prior stroke or TIA, index stroke severity by NIHSS, blood pressure on admission, symptoms (unilateral weakness, speech impairment) of the qualifying event, and results of laboratory tests and neuroimaging examinations (brain structure and vascular imaging) at admission and/or during hospitalization. The primary outcome was defined as a composite outcome of stroke, ACS and death from cardiovascular causes within 1 year. We investigated the associations between NLR and the primary outcome, in univariate and multivariate analyses, among all patients, and in a subgroup of patients aged ≥60 years (older patients).

### Laboratory Tests

All patients had a full blood cell counts test within 24 h after admission. The leukocyte, neutrophil and lymphocyte counts were retrieved from patients’ medical records. NLR was calculated by dividing the absolute neutrophil count by the absolute lymphocyte count. NLR was dichotomized by the 4th quartile, with NLR ≥ the 4th quartile as a high NLR. Fasting total cholesterol, triglycerides, high-density lipoprotein cholesterol (HDL-C), low-density lipoprotein cholesterol (LDL-C), and glucose levels were retrieved from the medical records during hospitalization. In addition, the glucose level upon admission was also collected.

### Neuroimaging Examinations

We recorded presence of new infarct(s) on brain CT or DWI, as well as presence of extra- and/or intra-cranial arterial stenosis on vascular imaging, conducted during hospitalization. Extracranial artery stenosis was defined as ≥50% lumen diameter reduction in carotid artery or vertebral artery on carotid duplex ultrasound or computed tomography angiography (CTA) by the NASCET method. Intracranial arterial stenosis (ICAS) was defined as ≥50% lumen diameter reduction in the intracranial portion of internal carotid artery, middle cerebral artery (M1/M2), anterior cerebral artery (A1/A2), posterior cerebral artery (P1/P2), intracranial portion of vertebral artery and basilar artery on magnetic resonance angiography (MRA) or CTA using the WASID method, or defined according to the criteria described in our previous study by transcranial Doppler (Wong et al., 2000).

### Follow-Up and Outcomes

All patients were treated according to latest guidelines, with antiplatelet or anticoagulant, and risk factor management, who were regularly followed up for 1 year at an outpatient clinic and treated as clinically indicated. The primary outcome was defined as a composite of stroke (either ischemic or hemorrhagic), ACS and vascular death within 1 year. Ischemic stroke was defined as a new symptomatic neurologic deterioration of vascular origin lasting more than 24 h, or a new symptomatic neurologic deterioration with new brain infarction on CT or magnetic resonance imaging (MRI) (Wang et al., 2013). Hemorrhagic stroke was defined as acute extravasation of blood into the brain parenchyma with associated neurologic symptoms (Wang et al., 2013). ACS included unstable angina and myocardial infarction with or without ST-segment elevation on electrocardiogram. Vascular death included fatal stroke, fatal ACS and sudden death (Amarenco et al., 2016). Occurrence of the outcome events was confirmed by referring to the clinical records.

### Statistical Analyses

Statistical analyses were conducted in all patients recruited, and in a subgroup of older patients (aged ≥60 years) as well. Continuous variables were expressed with medians (interquartile range, IQR), and categorical variables with numbers (percentage). Baseline data were compared between those with or without a primary outcome, using Chi-square tests or Fisher’s exact tests for categorical variables, and independent *t* tests or Mann–Whitney *U* tests for continuous variables. NLR was analyzed as continuous and binary variables in univariate analyses. Multivariate logistic regression analyses were conducted for the independent associations between dichotomized NLR (by the 4th quartile) and the primary outcome, adjusting for variables with *p* < 0.10 in univariate comparisons. Adjusted odds ratios (ORs) and the 95% confidence intervals (CI) were obtained. Two-sided *p* < 0.05 was considered statistically significant. All data were analyzed in IBM SPSS Statistics version 22.0 (SPSS Inc., Chicago, IL, United States).

## Results

### Characteristics of Patients

**FIGURE 1:**
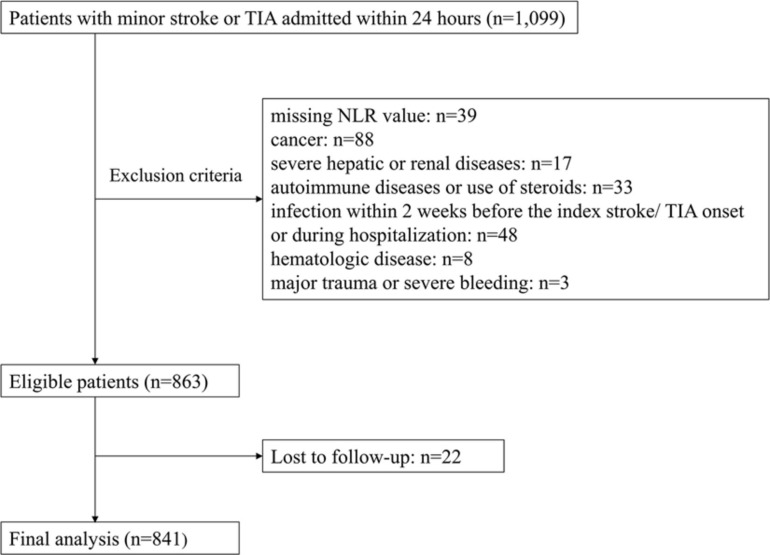
Study flow chart. NLR indicates neutrophil to lymphocyte ratio. TIA indicates transient ischemic attack.

From January 2011 to December 2015, a total of 1,099 patients with acute minor stroke or TIA were admitted within 24 h of symptom onset. Overall, 841 patients were analyzed in the current study, after excluding 39 patients with missing NLR value, 88 patients with a history of malignancy, 17 patients with severe hepatic or renal diseases, 33 patients with autoimmune diseases or use of steroids, 48 patients with known infection, 8 patients with hematologic diseases, 3 patients with major trauma or severe bleeding and 22 patients lost to follow-up (Figure 1).

Table 1 shows baseline characteristics of the 841 minor stroke or TIA patients enrolled in this study. The median age was 68 (IQR 59−76) years, 508 (60.4%) patients were male and 333 (39.6%) patients were female. Overall, the median counts of white blood cell, neutrophil and lymphocyte were 7.4 × 109/L (IQR 6.2−9.0 × 109/L), 4.7 × 109/L (IQR 3.8−6.0 × 109/L) and 1.8 × 109/L (IQR 1.3−2.3 × 109/L), respectively. The median NLR was 2.63 (IQR 1.89−3.83); 210 (25.0%) patients had high NLR (≥3.83).

**TABLE 1:** Characteristics of the patients at baseline*.

**Characteristics**	**Overall (*N* = 841)**
Age, years	68 (59−76)
Male	508 (60.4)
Ever smoking	324 (38.5)
**Medical history**	
Hypertension	565 (67.2)
Diabetes	214 (25.4)
Dyslipidemia	414 (49.2)
Atrial fibrillation	119 (14.1)
Ischemic heart disease	70 (8.3)
Prior ischemic stroke or TIA	178 (21.2)
Systolic blood pressure at admission, mmHg	161 (143−183)
Diastolic blood pressure at admission, mmHg	85 (75−97)
NIHSS at admission	1 (0−2)
Unilateral weakness	446 (53.0)
Speech impairment	302 (35.9)
Glucose at admission, mmol/L	6.6 (5.7−8.4)
**Laboratory test results during hospitalization**	
White blood cell, 109/L	7.4 (6.2−9.0)
Neutrophil, 109/L	4.7 (3.8−6.0)
Lymphocyte, 109/L	1.8 (1.3−2.3)
NLR	2.63 (1.89−3.83)
NLR ≥4th quartile (high NLR)	210 (25)
Fasting glucose, mmol/L	5.4 (5.0−6.2)
Triglycerides, mmol/L	1.2 (0.9−1.7)
Total cholesterol, mmol/L	4.7 (4.0−5.6)
HDL-C, mmol/L	1.3 (1.0−1.6)
LDL-C, mmol/L	2.8 (2.2−3.5)
HbA1c, %	6.1 (5.7−6.6)
**Neuroimaging results**	
New infarct(s)	377 (44.8)
Carotid stenosis	73 (10.4)
Intracranial arterial stenosis	223 (30.3)

*Values are medians (interquartile range) or numbers (%).NIHSS, National Institute of Health Stroke Scale; TIA, transient ischemic attack; NLR, neutrophil to lymphocyte ratio; HDL-C, high-density lipoprotein cholesterol; LDL-C, low-density lipoprotein cholesterol; HbA1c, glycosylated hemoglobin.

### Association Between NLR and the Primary Outcome in the Overall Cohort

Overall, 94 (11.2%) patients had a primary outcome within 1 year, including 71 non-fatal ischemic strokes, 5 non-fatal hemorrhagic strokes, 9 non-fatal ACSs, and 9 vascular deaths (2 ACSs, 1 hemorrhagic stroke, and 6 sudden deaths).

**FIGURE 2:**
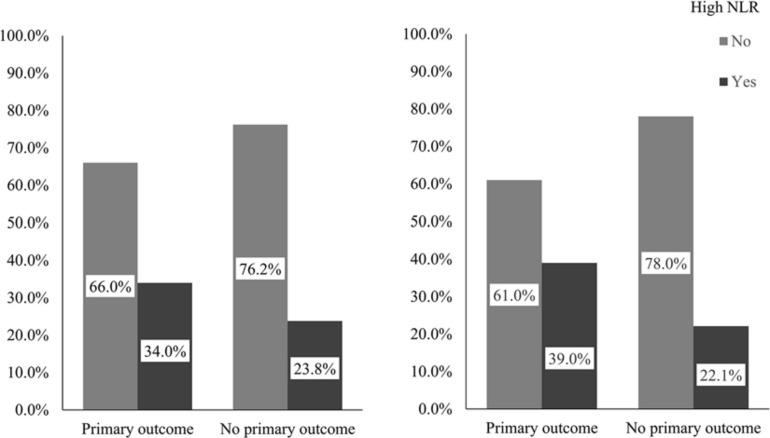
Percentages of patients with high NLR in those with or without a primary outcome. The left panel demonstrates percentages of patients with high NLR in those with or without a primary outcome in the overall cohort and the right in older patients. The primary outcome was a composite outcome of stroke (either ischemic or hemorrhagic), acute coronary syndrome and vascular death within 1 year. NLR indicates neutrophil to lymphocyte ratio.

Patients with a primary outcome were older (*p* < 0.001) and had lower diastolic blood pressure (*p* = 0.025) and higher glucose level at admission (*p* = 0.032); and more of them had a history of hypertension (*p* = 0.006) and diabetes (*p* = 0.023), presence of new infarct(s) on brain CT/MR imaging (*p* = 0.009) and presence of ICAS (*p* < 0.001), than those without a primary outcome. Moreover, the neutrophil levels (medians 4.9 versus 4.6; *p* = 0.032) and NLRs (medians 2.80 versus 2.60; *p* = 0.027) were significantly higher in patients with a primary outcome than those without; and more of those with a primary outcome had a high NLR (34.0% versus 23.8%; *p* = 0.031) (Figure 2). Other characteristics of the patients at baseline were not significantly different between patients with or with a primary outcome in univariate analyses (Table 2).

**TABLE 2:** Predictors of the primary outcome among all patients *.

**Characteristics**	**Primary outcome†**			**Multivariate logistic regression**	
	**No (*N* = 747)**	**Yes (*N* = 94)**	***P* value**	**Adjusted OR (95% CI)**	***P* value**
Age, years	67 (58−76)	73 (64−81)	<0.001	1.03 (1.01−1.06)	0.020
Every 10-year increment				1.37 (1.05−1.79)	
Male	448 (60.0)	60 (63.8)	0.471	1.00 (0.98−1.01)	0.615
Ever smoking	283 (37.9)	41 (43.6)	0.282		
**Medical history**					
Hypertension	490 (65.6)	75 (79.8)	0.006	1.25 (0.68−2.30)	0.478
Diabetes	181 (24.2)	33 (35.1)	0.023	1.32 (0.68−2.54)	0.412
Dyslipidemia	369 (49.4)	45 (47.9)	0.780		
Atrial fibrillation	100 (13.4)	19 (20.2)	0.074	1.74 (0.77−3.94)	0.184
Ischemic heart disease	59 (7.9)	11 (11.7)	0.208		
Prior ischemic stroke or TIA	152 (20.3)	26 (27.7)	0.102		
Systolic blood pressure at admission, mmHg	161 (144−182)	156 (138−185)	0.197		
Diastolic blood pressure at admission, mmHg	85 (75−98)	81 (72−90)	0.025		
NIHSS at admission	1 (0−2)	1 (0−3)	0.073	1.06 (0.84−1.34)	0.626
Unilateral weakness	396 (53.0)	50 (53.2)	0.974		
Speech impairment	268 (35.9)	34 (36.2)	0.955		
Glucose at admission, mmol/L	6.6 (5.6−8.4)	6.9 (6.0−9.0)	0.032	0.98 (0.90−1.06)	0.599
**Laboratory test results during hospitalization**					
White blood cell, 109/L	7.4 (6.2−8.9)	7.6 (6.4−9.3)	0.150		
Neutrophil, 109/L	4.6 (3.7−5.9)	4.9 (4.0−6.5)	0.032		
Lymphocyte, 109/L	1.8 (1.3−2.3)	1.6 (1.3−2.3)	0.352		
NLR	2.60 (1.86−3.73)	2.80 (2.19−4.86)	0.027		
NLR ≥4th quartile (high NLR)	178 (23.8)	32 (34.0)	0.031	1.42 (0.80−2.50)	0.229
Fasting glucose, mmol/L	5.4 (5.0−6.2)	5.5 (5.0−6.4)	0.423		
Triglycerides, mmol/L	1.2 (0.9−1.7)	1.2 (0.9−1.6)	0.363		
Total cholesterol, mmol/L	4.8 (4.0−5.6)	4.6 (3.8−5.3)	0.077	0.99 (0.76−1.28)	0.918
HDL-C, mmol/L	1.3 (1.0−1.6)	1.2 (1.0−1.5)	0.065	0.42 (0.20−0.91)	0.029
LDL-C, mmol/L	2.8 (2.2−3.5)	2.6 (2.0−3.5)	0.295		
HbA1c,%	6.1 (5.8−6.6)	6.1 (5.7−7.0)	0.736		
**Neuroimaging results**					
New infarct(s)	323 (43.2)	54 (57.4)	0.009	1.99 (1.15−3.44)	0.013
Carotid stenosis	64 (10.2)	9 (12.0)	0.637		
Intracranial arterial stenosis	181 (27.5)	42 (54.5)	<0.001	2.55 (1.51−4.29)	<0.001

*Values are medians (interquartile range) or numbers (%). Independent *t* tests or Mann−Whitney *U* tests were used for comparison of continuous variables. Chi-square tests or Fisher’s exact tests were used for comparison of categorical variables.†The primary outcome was a composite outcome of stroke (either ischemic or hemorrhagic), acute coronary syndrome and vascular death within 1 year.NIHSS, National Institute of Health Stroke Scale; TIA, transient ischemic attack; NLR, neutrophil to lymphocyte ratio; HDL-C, high-density lipoprotein cholesterol; LDL-C, low-density lipoprotein cholesterol; HbA1c, glycosylated hemoglobin; OR, odds ratio.

In multivariate logistic regression analysis in the overall cohort, older age, lower HDL-C level, and presence of new infarct(s) and ICAS, but not high NLR (adjusted OR 1.42; 95%CI 0.80−2.50; *p* = 0.229), were independently associated with the primary outcome (Table 2).

### High NLR as an Independent Predictor for the Primary Outcome in Older Patients

In this cohort, 612 patients aged ≥60 years (older patients), with a median age of 73 (IQR 66−79), 362 (59.2%) being male, 250 (40.8%) being female, and 229 aged <60 years, with a median age of 53 (IQR 48−57), 146 (63.8%) being male and 83 (36.2%) being female. The median white blood cell, neutrophil and lymphocyte counts among the older patients were 7.2 × 109/L (IQR 6.1−8.8 × 109/L), 4.6 × 109/L (IQR 3.7−5.9 × 109/L) and 1.7 × 109/L (IQR 1.3−2.2 × 109/L), respectively; the median NLR was 2.76 (IQR 1.96−4.00) and 148 (24.2%) had a high NLR.

Among older patients, 77 (13.5%) had a primary outcome within 1 year, 56 non-fatal ischemic strokes, 3 non-fatal hemorrhagic strokes, 9 non-fatal ACSs and 9 vascular deaths. Univariate analyses showed that older age (*p* = 0.002), history of hypertension (*p* = 0.008), lower HDL-C (*p* = 0.041), presence of new infarct(s) on brain imaging (*p* = 0.018) and ICAS (*p* < 0.001) were significantly associated with the primary outcome. Among older patients, those with a primary outcome had a significantly higher level of neutrophil (medians 4.8 versus 4.5; *p* = 0.036) and NLR (medians 2.96 versus 2.69; *p* = 0.007) at baseline than those without a primary outcome. Moreover, more of those with a primary outcome had a high NLR at baseline (39.0% versus 22.1%; *p* = 0.001) (Figure 2). Other baseline characteristics were not significantly different between those with and without a primary outcome among the 612 older patients (Table 3).

**TABLE 3:** Predictors of the primary outcome among older patients (aged ≥60 years) *.

	**All older patients**	**Primary outcome†**			**Multivariate logistic regression**	
	***N* = 612**	**No (*N* = 535)**	**Yes (*N* = 77)**	***P* value**	**Adjusted OR (95% CI)**	***P* value**
Age, years	73 (66−79)	73 (66−78)	76 (69−82)	0.002	1.06 (1.02−1.10)	0.008
Every 10-year increment					1.74 (1.16−2.61)	
Male	362 (59.2%)	314 (58.7)	48 (62.3)	0.543		
Ever smoking	235 (38.4%)	203 (37.9)	32 (41.6)	0.542		
**Medical history**						
Hypertension	448 (73.2)	382 (71.4)	66 (85.7)	0.008	1.56 (0.74−3.29)	0.247
Diabetes	168 (27.5%)	142 (26.5)	26 (33.8)	0.184		
Dyslipidemia	323 (52.8%)	282 (52.7)	41 (53.2)	0.930		
Atrial fibrillation	103 (16.8)	88 (16.4)	15 (19.5)	0.506		
Ischemic heart disease	61 (10.0)	51 (9.5)	10 (13.0)	0.344		
Prior ischemic stroke or TIA	139 (22.7%)	117 (21.9)	22 (28.6)	0.189		
Systolic blood pressure at admission, mmHg	161 (144−181)	161 (145−181)	158 (139−181)	0.266		
Diastolic blood pressure at admission, mmHg	82 (72−92)	83 (73−93)	80 (70−88)	0.095	0.99 (0.97−1.01)	0.370
NIHSS at admission	1 (0−2)	1 (0−2)	1 (1−2)	0.313		
Unilateral weakness	328 (53.6)	288 (53.8)	40 (51.9)	0.757		
Speech impairment	230 (37.6)	202 (37.8)	28 (36.4)	0.813		
Glucose at admission, mmol/L	6.7 (5.7−8.7)	6.7 (5.7−8.7)	6.9 (6.1−9.0)	0.108		
**Laboratory test results during hospitalization**						
White blood cell, 109/L	7.2 (6.1−8.8)	7.2 (6.1−8.7)	7.3 (6.2−9.2)	0.233		
Neutrophil, 109/L	4.6 (3.7−5.9)	4.5 (3.6−5.8)	4.8 (4.0−6.8)	0.036		
Lymphocyte, 109/L	1.7 (1.3−2.2)	1.7 (1.3−2.2)	1.5 (1.2−2.1)	0.127		
NLR	2.76 (1.96−4.00)	2.69 (1.92−3.84)	2.96 (2.34−5.10)	0.007		
NLR ≥4th quartile (high NLR)	148 (24.2%)	118 (22.1)	30 (39.0)	0.001	2.00 (1.07−3.75)	0.031
Fasting glucose, mmol/L	5.5 (5−6.3)	5.5 (5−6.2)	5.5 (5.1−6.4)	0.789		
Triglycerides, mmol/L	1.2 (0.9−1.6)	1.2 (0.9−1.6)	1.1 (0.9−1.5)	0.453		
Total cholesterol, mmol/L	4.7 (4.0−5.5)	4.7 (4.0−5.5)	4.5 (3.7−5.3)	0.172		
HDL-C, mmol/L	1.3 (1.0−1.6)	1.3 (1.0−1.6)	1.2 (1.0−1.5)	0.041	0.30 (0.13−0.68)	0.004
LDL-C, mmol/L	2.7 (2.1−3.4)	2.7 (2.1−3.4)	2.6 (2.0−3.5)	0.577		
HbA1c,%	6.1 (5.8−6.6)	6.1 (5.8−6.6)	6.1 (5.7−6.8)	0.815		
**Neuroimaging results**						
New infarct(s)	265 (43.3)	222 (41.5)	43 (55.8)	0.018	2.40 (1.29−4.46)	0.006
Carotid stenosis	68 (13.8)	60 (13.8)	8 (13.6)	0.961		
Intracranial arterial stenosis	165 (31.6)	132 (28.6)	33 (54.1)	<0.001	2.42 (1.35−4.35)	0.003

*Values are medians (interquartile range) or numbers (%); Independent *t* tests or Mann−Whitney *U* tests were used for comparison of continuous variables. Chi-square tests or Fisher’s exact tests were used for comparison of categorical variables.†The primary outcome was a composite outcome of stroke (either ischemic or hemorrhagic), acute coronary syndrome and vascular death within 1 year.NIHSS, National Institute of Health Stroke Scale; TIA, transient ischemic attack; NLR, neutrophil to lymphocyte ratio; HDL-C, high-density lipoprotein cholesterol; LDL-C, low-density lipoprotein cholesterol; HbA1c, glycosylated hemoglobin; OR, odds ratio.

Among older patients, a high NLR (adjusted OR 2.00; 95%CI 1.07−3.75; *p* = 0.031) was independently associated with the primary outcome, adjusted for confounders in multivariate logistic regression (Table 3). In addition, older age (adjusted OR 1.74 for every 10-year increment; 95%CI 1.16−2.61; *p* = 0.008), lower HDL-C level (adjusted OR 0.30; 95%CI 0.13−0.68; *p* = 0.004), presence of new infarct(s) on brain imaging (adjusted OR 2.40; 95%CI 1.29−4.46; *p* = 0.006) and ICAS (adjusted OR 2.42; 95%CI 1.35−4.35; *p* = 0.003) were also independently associated with increased risk of recurrent vascular events within 1 year in older patients (Table 3).

## Discussion

In the current study, we found that a high NLR was independently associated with an increased risk of any vascular event within 1 year among older patients (aged ≥60 years) with a minor stroke or TIA admitted within 24 h. Specifically, older minor stroke or TIA patients with a NLR in the highest quartile (versus less than the 4th quartile) had a doubled risk of a combined outcome of recurrent stroke, myocardial infarction or vascular death. However, NLR was not independently associated with the 1-year risk of vascular events in overall analyses in TIA/minor stroke patients.

Previous studies have indicated an independent predictive role of elevated NLR for risk of stroke in both healthy subjects and ischemic stroke patients. For instance, elevated NLR (adjusted hazard ratio [HR] for NLR ≥3.5: 2.96; 95%CI 1.57−5.58; *p* < 0.001) was independently associated with a long-term risk of ischemic stroke in healthy adults (median follow-up = 5.9 years) (Suh et al., 2017). The study indicated that NLR was more predictive of ischemic stroke than C-reactive protein, suggesting that NLR and C-reactive protein may represent different biological aspects of inflammation. NLR (adjusted HR 1.50; 95% CI 1.16−1.94; *p* = 0.002) was also significantly related to recurrent ischemic stroke (median follow-up = 1.13 years) among ischemic stroke patients, after adjusting for conventional cardiovascular risk factors (Xue et al., 2017).

In addition, NLR had a significantly positive association with increased carotid intima–media thickness (IMT) in male patients with ischemic stroke (Hyun et al., 2015). Another group also reported that NLR ≥2.6 was an independent predictor for carotid artery stenosis to become symptomatic (Köklü et al., 2016). Moreover, NLR ≥1.52 was predictive of ICAS in healthy Korean subjects, and the relationship between elevated NLR and the number of ICAS lesions was in a dose-dependent manner (Nam et al., 2018). Both carotid (including increased IMT and carotid stenosis) and intracranial atherosclerosis have been identified as significant risk factors of stroke and all cardiovascular events in previous studies. In the current study, ICAS was an independent predictor of vascular events in overall analyses and in older patients, while carotid stenosis was not, possibly due to the small number of patients with carotid stenosis in this cohort.

The mechanisms linking NLR to extra- and intracranial atherosclerosis and risk of ischemic strokes have not yet been fully clarified, but the following might provide some insights. The important role of inflammatory responses has been well established in pathogenesis of ischemic stroke. Neutrophils are the first blood-borne immune cells to arrive at ischemic brain tissues. Possible effects of neutrophils in stroke include damaging brain tissue by releasing reactive oxygen species and pro-inflammatory cytokines, initiating thrombosis by endothelial activation and restricting the cerebral blood flow (Ramiro et al., 2018). Additionally, neutrophils could also induce arterial thrombosis and subsequent brain ischemia by increasing the instability of atherosclerotic plaques via production of matrix metallopeptidase 9, or forming neutrophil extracellular traps (basically composed of DNA from neutrophils) that may lead to thrombus formation through recruitment of red blood cells and facilitation of fibrin deposition (Ionita et al., 2010; Sørensen and Borregaard, 2016).

On the other hand, regulatory T cells, a subtype of lymphocytes, play a major protective role against neuroinflammation in ischemic stroke. Depletion of these cells profoundly increased brain injuries and worsen functional outcomes after an acute ischemic stroke in animal models (Liesz et al., 2009). Moreover, lower lymphocyte counts were observed to be significantly associated with poor neurologic improvement within the first week, and with unfavorable functional outcome at 3 months, among ischemic stroke patients (Kim et al., 2012). Relative lymphopenia aggravated ischemic injuries with decreased production of anti-inflammatory cytokines, such as interleukin 10 (Liesz et al., 2015). Therefore, NLR, an index representing the relative counts of neutrophil and lymphocyte, more comprehensively reflects inflammatory responses after cerebral ischemia than either neutrophils or lymphocytes alone.

Of note, aged brain is more vulnerable to ischemia insult. In experimental ischemic stroke models, a decrease of claudin-5 and zonula occludens-1 (ZO-1) (markers of blood brain barrier), more Evans blue leakage and an increase of interleukin-6 (IL-6), interleukin-1β (IL-1β), and C-C Motif Chemokine Ligand 2 (CCL2) (proinflammatory chemokine), were observed in aged rats, compared with their younger counterparts (DiNapoli et al., 2010; Shen et al., 2019). Significant increase in lipid peroxidation and lipofuscin contents, decreased activity of antioxidant enzymes and a lower level of Na^+^, K^+^-ATPase performance have been found in aged brain (Scavone et al., 2005; Bala et al., 2006). Moreover, neurotrophic factors, such as brain-derived neurotrophic factor and basic fibroblast growth factor, significantly decrease in both the cortex and striatum in old mice as compared with young mice after stroke, leading to impaired support to the neurons at risk of death from ischemia (Chen and Sun, 2007; Arumugam et al., 2010). Taken together, these alterations in aged brain makes it more susceptible to inflammatory injury when cerebral ischemia occurs, which might partly explained findings in the current study that NLR was independently associated with higher risk of vascular events (mostly ischemic stroke) only in older patients.

In addition to the stroke risks, accumulative evidence also showed association between elevated NLR and increased risk of developing significant coronary artery disease (Kaya et al., 2014; Verdoia et al., 2016). Besides, elevated NLR may also increase the risks of cardio- and cerebrovascular diseases via its correlations with vascular risk factors. For instance, a study of 28,850 healthy subjects indicated that NLR in the highest quintile was independently associated with a higher risk of developing hypertension with a median follow-up of 2.63 years (adjusted HR 1.23 for NLR in the highest versus lowest quintiles) (Liu et al., 2015). Moreover, diabetic patients had significantly higher NLR than non-diabetic patients (means: 3.4 versus 3.1; *p* = 0.004), in a study of 3,756 patients undergoing coronary angiography (Verdoia et al., 2015). Therefore, a higher NLR, as a marker of systemic inflammation, was associated with all vascular events in older patients in the current cohort.

There are several limitations of the present study. First, this was a single-center study, and with strict exclusion criteria, 258 (23.5%) patients were excluded among 1,099 patients, which might affect the generalizability of our findings. However, most patients were excluded due to factors that might significantly affect the systemic inflammation status and the NLR value, which would have interfered with the current analyses. Second, we only collected NLR data in one snapshot after the index stroke/TIA, although it changes over time. A previous study showed an increasing trend in the NLR 48 h after thrombolysis in patients with ischemic stroke and NLR at 12–18 h might provide most predictive value in identifying hemorrhagic transformation after thrombolysis. Thus, clinical significance of temporal profile of NLR following the index stroke/TIA warrant further investigations (Guo et al., 2016). Third, we excluded patients with underlying conditions that may significantly affect NLR, but some factors that might affect risk of stroke recurrence and other vascular events, such as CRP, obesity, amount of exercise, were not adjusted in multivariate analyses of the association between NLR and vascular events in this study. Further, we did not adjust for treatment methods of the patients in the multivariate analyses, while all patients received routine treatment according to latest guidelines, which may not significantly interfere with the study findings. Last, stroke subtype and infarct patterns might affect the risk of stroke relapse, which were not analyzed in the current study. Further prospective investigations in different populations are warranted, for the effects of dynamic NLR levels in governing the risk of stroke and other vascular events in healthy subjects and stroke patients.

## Conclusion

Neutrophil to lymphocyte ratio, a marker of systemic inflammation that can be easily obtained in routine blood tests, might predict recurrent vascular events in older (e.g., aged ≥60 years) patients with minor stroke or TIA. This may provide new therapeutic target for neuroprotection after an ischemic stroke or TIA, to improve the functional outcome and reduce the risk of recurrence. Further studies are warranted to verify the current findings in larger prospective cohorts in different populations, with more NLR measures after an index stroke or TIA to reflect the dynamic change in NLR levels.

## Data Availability Statement

The raw data supporting the conclusions of this article will be made available by the authors, without undue reservation.

## Ethics Statement

Ethical review and approval was not required for the study on human participants in accordance with the local legislation and institutional requirements. Written informed consent for participation was not required for this study in accordance with the national legislation and the institutional requirements.

## Author Contributions

KC, XF, TL, and XL contributed to the conception and design of the study. KC, XF, and XL contributed to drafting the text and preparing the figures. All authors contributed to the acquisition and analysis of data.

## Conflict of Interest

The authors declare that the research was conducted in the absence of any commercial or financial relationships that could be construed as a potential conflict of interest. The reviewer YW declared a shared affiliation, with no collaboration, with one of the authors XF to the handling editor at the time of the review.

## Abbreviations

ACS: acute coronary syndrome
AF: atrial fibrillation
CT: computed tomography
CTA: computed tomography angiography
DWI: diffusion-weighted magnetic resonance imaging
HDL-C: high-density lipoprotein cholesterol
HR: hazard ratio
ICAS: intracranial arterial stenosis
IMT: intima–media thickness
IQR: interquartile range
LDL-C: low-density lipoprotein cholesterol
MRA: magnetic resonance angiography
MRI: magnetic resonance imaging
NIHSS: National Institute of Health Stroke Scale
NLR: neutrophil to lymphocyte ratio
OR: odds ratio
TIA: transient ischemic attack.
